# Date Seeds Flour Used as Value-Added Ingredient for Wheat Sourdough Bread: An Example of Sustainable Bio-Recycling

**DOI:** 10.3389/fmicb.2022.873432

**Published:** 2022-04-18

**Authors:** Hana Ameur, Vincenzo Cantatore, Pasquale Filannino, Ivana Cavoski, Olga Nikoloudaki, Marco Gobbetti, Raffaella Di Cagno

**Affiliations:** ^1^Faculty of Science and Technology, Libera Università di Bolzano, Bolzano, Italy; ^2^Department of Soil, Plant and Food Science, University of Bari Aldo Moro, Bari, Italy; ^3^CIHEAM-MAIB, Mediterranean Agronomic Institute of Bari, Valenzano, Bari, Italy

**Keywords:** date-seeds, fermentation, lactic acid bacteria, yeasts, phenolics, sourdough bread, bio recycling

## Abstract

Our study proposed date seeds flour (DSF) as an innovative ingredient for sourdough bread production through sustainable bio-recycling. We isolated autochthonous lactic acid bacteria and yeasts from DSF and DSF-derived doughs to build up a reservoir of strains from which to select starters ensuring rapid adaptation and high ecological fitness. The screening based on pro-technological criteria led to the formulation of a mixed starter consisting of *Leuconostoc mesenteroides*, *Lactiplantibacillus plantarum*, and *Saccharomyces cerevisiae* strains, which allowed obtaining a mature type I sourdough after consecutive refreshments, in which an aliquot of the durum wheat flour (DWF) was replaced by DSF. The resulting DSF sourdough and bread underwent an integrated characterization. Sourdough biotechnology was confirmed as a suitable procedure to improve some functional and sensory properties of DWF/DSF mixture formulation. The radical scavenging activity increased due to the consistent release of free phenolics. Perceived bitterness and astringency were considerably diminished, likely because of tannin degradation.

## Introduction

Bread is an ancient staple food still representing one of the most popular meals consumed worldwide. In the current conception of the food system, some authors reevaluated the potential of bread to cope with future food supply challenges, although some issues remain to be addressed, such as the sustainability of the supply chain, the nutrients coverage, and digestibility of bread (Weegels, 2019; Thielecke et al., 2021). In this scenario, the development of approaches to sustainably hybridize wheat flour with others deriving from non-wheat cereals, pseudo-cereals, pulses, oily seeds, non-grain plants, or agro-food by-products is challenging (Gobbetti et al., 2020). This hybridization of alternative ingredients may represent a valuable source of nutrients and micronutrients (e.g., proteins and minerals) or health-promoting components (e.g., phytochemicals and fibers). Focusing on supply chain sustainability and recycling of wastes, we proposed to use date seeds flour (DSF) as a value-added ingredient for wheat sourdough bread making. During the last decade, the cultivation of the date palm has gone beyond the boundaries of its traditional production area, nearly doubling the world production, which achieved *ca.* 8.5 million tons in 2018 (Echegaray et al., 2021). Date seeds constitute an abundant residue of the pitted date industry, as they represent up to 15% of the fruit mass, which are mostly discarded as wastes or used as feeding supplements (Echegaray et al., 2021). This lack of interest conflicts with the high content of valuable compounds in date seeds, which include fiber, unsaturated fatty acids, proteins, phenolic compounds, phytosterols, tocopherols, tocotrienols, carotenes, and minerals (Habib et al., 2013; Wahini, 2016; Hilary et al., 2020; Echegaray et al., 2021). Some authors hypothesized that DSF proteins might originate bioactive peptides and associated DSF to antioxidant and ACE inhibitory activities (Ambigaipalan et al., 2015; Saeed et al., 2021). Nevertheless, the addition of non-wheat flours in baked good formulas might lead to losses of acceptability, because of the worsening of sensory and rheology attributes. The high level of fiber in DSF (65–69%) may result in breads with poor specific volume and softness, due to its interference with the gluten network and the lowered gas-holding capacity of the dough (Rosell and Santos, 2010). Furthermore, DSF is rich in tannins, which cause an important increase in astringency perception (Echegaray et al., 2021). Consequently, the manufacture of fortified baked goods without compromising their sensory appeal is the main challenge for researchers and the food industry (Sandvik et al., 2018).

Biological processes have always been central elements in bread production, where sourdough fermentation was recognized as an effective biotechnology to leverage the technological, nutritional, and sensory features of agro-food side streams in bread making (Arora et al., 2021). Sourdough fermentation of non-conventional flours offers the opportunity of increasing the bioavailability of nutrients and functional compounds and decreasing the level of antinutritional factors like condensed tannins and phytic acid. Fermentation processes depend on specific determinants, with the flour chemical composition being one of the most important determinants. In fact, the level and type of fermentable carbohydrates, nitrogen sources, and other growth factors affect the sourdough microbial dynamics. On the contrary, the high levels of phenolics in DSF may limit the microbial growth, including that of lactic acid bacteria (Rodríguez et al., 2009; Sánchez-Maldonado et al., 2011; Filannino et al., 2014; Dinardo et al., 2019), which are the main players in sourdough along with yeasts. Consequently, suitable starter cultures should be selected to valorize the potential of DSF as an ingredient in wheat sourdough bread under controlled conditions, ensuring adequate metabolic performances and improved nutritional, functional, and sensory properties (Minervini et al., 2014; Gänzle and Zheng, 2019; Xu et al., 2019). With this perspective, the definition of the optimal ratio between DSF and wheat flour represents a critical point for DSF exploitation.

To the best of our knowledge, the effects of DSF as a sourdough ingredient on microbial dynamics and the fermentation performances have not been investigated to date. First, our study aimed at characterizing autochthonous lactic acid bacteria and yeasts according to the main pro-technological features (acidification and leavening capacities) and selecting a mixed starter to be used in DSF sourdough fermentation. The resulting DSF sourdough and bread underwent an integrated characterization. All results converged to define: optimal DSF use conditions; main microbiological and biochemical characteristics of DSF sourdough; and determinant microbial species for maximizing nutritional, functional, and sensory characteristics of DSF sourdough bread.

## Materials and Methods

### Flours

Organic date seeds were provided by the company Boudjebel SA VACPA, located in Tunisia. Dates (Deglet Nour cultivar), collected at the “Tamr” full ripeness stage from Tozeur and Kebili Tunisia regions, were pitted and processed as date paste. Seeds by-products were washed with tap water. Then, washed seeds were dried under the sun (*ca.* 40–45°C) for 4–5 days. After drying, seeds were grounded through two steps. In the first step, seeds were grounded into a coarse powder using a hammer mill, while the second step gave a fine powder by a heavy-duty grinder. Finally, the resulting DSF was sieved using a 0.5-mm sieve and stored at room temperature. Durum wheat flour (DWF) was purchased from an organic market in Italy.

### Date Seeds Flour and Durum Wheat Flour Proximate Analysis

Protein (total nitrogen * 6.25), fat, ash, and water content of DSF and DWF were determined according to the AOAC approved methods 978.04, 2003.06, 930.05, and 930.04, respectively. Total available carbohydrates were calculated as the difference [100 – (Proteins + Ash + Total fat + Water)]. Insoluble and soluble dietary fibers were determined according to the approved AOAC 991.432 and 993.19 methods, respectively. Energy value was calculated as described by the United States Department of Agriculture method (Institute of Medicine of the National Academies [Iom]., 2002).

### Isolation of Lactic Acid Bacteria and Yeasts

Lactic acid bacteria and yeasts were isolated from (i) DSF; (ii) DSF dough, having dough yield (DY) (dough weight × 100/flour weight) of 224 and incubated at 30°C for 16 h; and (iii) DSF type I sourdough. DSF type I sourdough was made and propagated through the wheat flour traditional protocol (Di Cagno et al., 2014), without the addition of starter cultures or baker’s yeast. DSF was mixed with tap water (60 rpm for 5 min) to produce a dough (DY of 224), which represents the first step prior to fermentation and before becoming sourdough. After the first fermentation lasting 16 h at 30°C, sourdough propagation was according to the backs-lopping (refreshment) procedure, where the sourdough from the day before was used as the starter [20% (w/w) of inoculum] to ferment (16 h at 30°C) a new mixture of flour and tap water, having a DY of 224. Eight refreshments were carried out. Between each daily fermentation, sourdoughs were stored at 4°C for *ca.* 16 h.

The pH value of sourdoughs was measured by a pH meter (Model 507, Crison, Milan, Italy) with a food penetration probe. Total titratable acidity (TTA) was determined after the homogenization of 10 g of sourdough with 90 ml of distilled water and expressed as the amount (ml) of 0.1 M NaOH required to neutralize the solution using phenolphthalein as an indicator (official AACC method 02-31.01). The increase in the volume of sourdoughs (ΔV, cm^3^) was calculated as the difference between the volume of the dough at the end and the beginning of fermentation (Minervini et al., 2011). After eight refreshments, the acidification rate and volume increase were stable, and DSF sourdough was used for the microbiological analysis and isolation of lactic acid bacteria and yeasts. Aiming to compensate any nutritional deficiencies for the growth of both lactic bacteria and yeasts, parallel dough fermentations were performed with the addition of (i) glucose (10 g/kg) and fructose (10 g/kg); (ii) yeast extract (5 g/kg); or (iii) glucose (10 g/kg), fructose (10 g/kg), and yeast extract (10 g/kg).

Ten grams of DSF, dough, sourdough, sourdough added with sugars, sourdough added with yeast extract, and sourdough added with sugars and yeast extract were homogenized with 90 ml of sterile peptone water (1%, w/v) and NaCl solution (0.9%, w/v). Serial dilutions were plated on different agar media. Presumptive lactic acid bacteria were enumerated using de Man, Rogosa and Sharpe (MRS) (Oxoid, Basingstoke, Hampshire, United Kingdom) and modified MRS (mMRS) containing maltose (1%, w/v) and fresh yeast extract (5%, w/v), adjusted to pH 5.6 (Oxoid). Media were supplemented with cycloheximide (0.1 g/L). Plates were incubated at 30°C for 48 h under anaerobiosis (AnaeroGen and AnaeroJar, Oxoid). Approximately ten colonies of presumptive lactic acid bacteria were randomly selected from the plates containing the highest sample dilutions. Gram-positive, catalase-negative, non-motile rods and cocci isolates were cultivated into MRS, or mMRS, broth at 30°C for 24 h and re-streaked onto the same agar medium until pure cultures were obtained. All isolates considered for further analyses were able to acidify the culture medium.

Yeasts were cultured onto Wallerstein Laboratory Nutrient Agar (Oxoid) with chloramphenicol (0.1 g/L) and incubated at 30°C for 96 h (Pallmann et al., 2001). Approximately five colonies of yeasts were picked up, based on the morphology, from the plates containing the highest sample dilutions. Isolates were subcultured in yeast extract-peptone-dextrose (YPD) broth (Sigma-Aldrich, Steinheim, Germany) and re-streaked onto the same agar medium.

### Typing and Identification of Lactic Acid Bacteria and Yeasts

Bacterial and yeast isolates were identified by partial sequencing of the 16S and 26S rRNA genes, respectively (Lhomme et al., 2015). In detail, genomic DNA was extracted from bacteria using DNeasy Blood and Tissue Kit (Qiagen, Valencia, CA), according to the manufacturer’s instructions. Two primer pairs, LacbF/LacbR and LpCoF/LpCoR (Sigma-Aldrich), were used to amplify the 16S rRNA gene fragment of lactic acid bacteria. Differentiation between *Lactiplantibacillus plantarum* and *Lactiplantibacillus pentosus* was carried out by partial sequencing of the *recA* gene (Torriani et al., 2001). Primer pairs NL-1/NL-4 (Sigma-Aldrich) were used to amplify the D1/D2 domain of the 26S rRNA gene of yeasts. PCR products were separated by electrophoresis. The expected amplicons were eluted from the gel, purified by the Nucleospin gel and PCR cleanup kit (Macherey-Nagel, Düren, Germany), and subjected to Sanger sequencing (Sanger et al., 1977). Identification was performed by comparing the sequences of each isolate with those reported in the Basic BLAST database (Altschul, 1997). Strains showing homology of at least 97% were considered to belong to the same species (Goebel and Stackebrandt, 1994). Gene sequences have been deposited in GenBank under the accession numbers OM731650-OM731662; OM731665-OM731670.

Bacterial and yeast isolates were also typed by randomly amplified polymorphic DNA-PCR (RAPD-PCR) analysis, using primers and conditions described by Lhomme et al. (2015). Three oligonucleotides, namely, P4, P7, and M13, with arbitrarily chosen sequences were used for typing of bacterial isolates. Primers M13m and RP11 were used for typing of yeasts. RAPD-PCR profiles were acquired by the MCE-202 MultiNA microchip electrophoresis system (Shimadzu Italia s.r.l., Milan, Italy) using the DNA-12000 reagent kit (100–12,000 bp) and the 2-log DNA ladder (0.1–10.0 kb) according to the manufacturer’s instructions. The similarity of the electrophoretic profiles was evaluated by the Pearson product-moment correlation coefficient (r) and using the unweighted pair group method arithmetic mean (UPGMA) analysis. The reproducibility of the RAPD profiles was assessed by comparing the PCR products obtained from DNA prepared from three independent cultures of the same isolate. Combined RAPD profiles were subjected to cluster analysis by the UPGMA analysis. Cultures were maintained as stocks in 15% (w/v) glycerol at −80°C.

### Selection of Autochthonous Lactic Acid Bacteria and Yeasts

Aiming to select lactic acid bacteria and yeast, a growth model system was used to investigate the kinetics of acidification of bacteria and the leavening capacity of yeasts. DSF (20%, w/w of flour) and wheat flour (DWF, 80% w/w of flour) were mixed with tap water to prepare 100 g of dough (dough yield of 174) representing the growth model system. The ratio between DSF and DWF was chosen to obtain a dough suitable for kneading and leavening.

Twenty-nine representative strains of lactic acid bacteria were propagated into MRS or mMRS broth (depending on the isolation media) at 30°C until the late exponential growth phase was reached (*ca.* 12 h). Cells were harvested by centrifugation (10,000 × *g*, 10 min at 4°C), washed twice in 50 mM sterile potassium phosphate buffer (pH 7.0), and resuspended into DSF dough to a final cell density corresponding to *ca.* 7.0 Log CFU/g. Mixing was done manually for 5 min. Doughs were incubated at 30°C for 13.5 h, according to the optimal growth temperature and the fermentation time of the selected lactic acid bacteria achieving the proper biochemical properties (Coda et al., 2010; Nionelli et al., 2014a). The value of pH was measured by a Crison pH meter (model 507; Crison). Kinetics of acidification was determined and modeled according to the Gompertz equation as modified by Zwietering et al. (1990): *y* = *k* + *A* exp{−exp[(*V*_max_ *e*/*A*)(λ− *t*) + 1]}, where *k* is the initial level of the dependent variable to be modeled (pH units), *A* is the difference in pH units (ΔpH) between inoculation and the stationary phase, *Vmax* is the maximum acidification rate (expressed as pH/h), λ is the length of the lag phase (expressed in hours), and *t* is the time. Aiming to investigate the synthesis of exo-polysaccharides (EPS) by autochthonous lactic acid bacteria, colonies from cell suspensions of each strain were allowed to grow on MRS agar with the addition of 292 mM sucrose, 146 mM glucose, or 146 mM fructose. After incubation at 30°C for 48 h, the synthesis of EPS was evaluated by the visual appearance of the mucoid colonies. Twenty-two representative yeast strains were propagated at 30°C in YPD broth, and cells at the late exponential growth (*ca.* 24 h) phase were washed two times in 50 mM phosphate buffer, pH 7.0, and used to inoculate DSF doughs to a final cell density corresponding to *ca.* 7.0 Log CFU/g. Doughs were incubated at 30°C for 13.5 h. During incubation, the increase in the volume was manually monitored every 2 h. Leavening performance was determined in terms of Δ*V*/*t* (cm^3^/h) (Minervini et al., 2011).

### Date-Seed Sourdough Fermentation and Bread-Making

Based on their acidification and leavening capacities, *Leuconostoc mesenteroides* DDL1, *Lactiplantibacillus plantarum* DCL9, and *Saccharomyces cerevisiae* DSNc1 and DDNd10 were chosen as selected mixed starters for DWF and DSF sourdough fermentation. DSF was used as an ingredient (20%, w/w of flour) for DWF substitution to produce the DWF/DSF sourdough. Cellular suspensions of starters were prepared as previously described. DSF (40 g) and DWF (160 g) were mixed with tap water (200 ml) containing the above cellular suspension (cell density in the dough of *ca.* 7.0 Log CFU/g) to obtain 400 g of dough (dough yield of 200). The dough was fermented at 30°C for 13.5 h. Then, DWF/DSF sourdough was daily propagated for 10 days according to a backslopping protocol to obtain a mature DWF/DSF type I sourdough. In detail, sourdough was added (inoculum rate of 20%, w/w) to a mixture of tap water and flour (ratio DSF:DWF of 20:80) to prepare a dough (DY of 200). DWF/DSF sourdough was fermented at 30°C for 13.5 h and stored at 4°C between back-slopping steps. A baker’s yeast-fermented dough containing the same percentage of DSF (20%, w/w of flour) but without the use of starters (DWF/DSF-baker’s yeast-fermented dough) and a baker’s yeast-fermented dough without the addition of DSF (DWF-baker’s yeast-fermented dough) were used as the controls. Commercial compressed baker’s yeast was used at the percentage of 2% w/w, and doughs were incubated at 30°C for 1.5 h, according to a protocol previously optimized to guarantee the best technological features of dough. Unfermented dough containing the same percentage of DSF (20%, ww of flour) and dough without the addition of DSF were also analyzed (DWF/DSF-unfermented dough and DWF-unfermented dough, respectively).

A DWF/DSF type I sourdough bread (DWF/DSF-SB) was manufactured at a pilot plant level. Control breads were also obtained from DWF/DSF-baker’s yeast-fermented dough (DWF/DSF-BYB) and DWF-baker’s yeast-fermented dough (DWF-BYB). All pieces of bread were baked (220°C for 40 min) in a rotary oven (Combo 3, Zucchelli, Verona, Italy).

### Microbiological and Chemical Analyses of Sourdoughs

Ten grams of sourdough were homogenized with 90 ml of sterile peptone water (1%, w/v) and NaCl solution (0.9%, w/v). Serial dilutions were plated on different agar media. Presumptive lactic acid bacteria were enumerated onto MRS and mMRS agar media (Oxoid) supplemented with cycloheximide (0.1 g/L) as described above. Yeasts were enumerated onto YPD agar medium (Sigma Chemical) supplemented with chloramphenicol (0.1 g/L) and incubated at 30°C for 96 h. Enterobacteria were enumerated on violet red bile glucose (VRBG) agar (Oxoid) plates and incubated at 37°C for 24 h. RAPD-PCR analysis monitored selected cultures during back-slopping steps.

Total titratable acidity and pH were monitored as described above. Water/salt-soluble extract (WSE) of doughs, prepared according to Weiss et al. (1993), was used to analyze organic acids, proteins, peptides, and total free amino acids (TFAAs). Organic acids were determined by high-performance liquid chromatography (HPLC), using an ÄKTA Purifier System (GE Healthcare, Buckinghamshire, United Kingdom) equipped with an Aminex HPX-87H column (ion exclusion, Bio-Rad, Richmond, CA, United States) and a UV detector operating at 210 nm. Elution was at 60^⋅^C, with a flow rate of 0.6 ml/min using H_2_SO_4_ 10 mM as mobile phase (Rizzello et al., 2010a). The quotient of fermentation (QF) was determined as the molar ratio between lactic and acetic acids. TFAAs were analyzed by a Biochrom 30 series Amino Acid Analyzer (Biochrom Ltd., Cambridge Science Park, United Kingdom) with a Na-cation-exchange column (20 by 0.46 cm internal diameter), as described by Rizzello et al. (2010a). For the peptides analysis, WSE was treated with trifluoroacetic acid (0.05% w/v) and subjected to dialysis (cutoff 500 Da) to remove proteins and FAA. Then, peptides concentration was determined by the o- phthalaldehyde (OPA) method as described by Church et al. (1983). The concentration of proteins in WSE was determined according to the Bradford method (Bradford, 1976).

Total free phenolic compounds were determined in methanol/water-soluble extracts. One gram of freeze-dried dough was resuspended in 9 ml of methanol:water (80:20). The mixture was purged under a nitrogen stream and stirring condition for 30 min and centrifuged at 4,600 × *g* for 20 min. Total free phenolic compounds were quantified as described by Slinkard and Singleton (1977) and expressed as mg gallic acid equivalent/g of dough.

### Antioxidant Activities of Sourdoughs

Antioxidant activity of sourdough was assayed as scavenging activity on 1,1-diphenyl-2-picrylhydrazyl radical (DPPH^⋅^) (Yu et al., 2003). The analyses were carried out using WSE and methanol-soluble extracts (MSE). For MSE preparation, 1 g of freeze-dried dough was resuspended in 9 ml of methanol acidified with HCl (0.1% v/v) and incubated at room temperature for 1 h under stirring condition (200 rpm). The supernatants were collected by centrifugation (11,000 × *g* for 20 min). The reaction mixture was prepared by diluting both WSE and MSE extracts in water and methanol, respectively. The reaction was monitored by reading the absorbance at 517 nm every 2 min for 30 min. A blank reagent was used to verify the stability of DPPH^⋅^ over the test time. Butylated hydroxytoluene (BHT) was used as the antioxidant reference.

### Technological and Proximal Characterization of Bread

The specific volume of bread was measured using the rapeseed displacement method (ACCI Method 10-05.01, Guidelines for Measurement of Volume by Rapeseed Displacement). In detail, the bread loaf was weighed and placed into a 2-L beaker. After having completely covered the bread by pouring rapeseeds in the beaker, the volume was measured (cm^3^). Then, the bread was removed from the beaker, and the volume of rapeseeds alone was measured. Specific volume (cm^3^/g) was calculated as the difference between the two measured volumes divided by bread weight. The crumb cells of bread were analyzed after 24 h of storage using the image analysis technology. Images of the sliced pieces of bread were captured using an Image Scanner (Amersham Pharmacia Biotech, Uppsala, Sweden). Images were scanned full-scale at 300 dots per inch and analyzed in grayscale (0–255). Image analysis was performed using the UTHSCSA ImageTool program (version 2.0, University of Texas Health Science Centre, San Antonio, Texas, United States). A threshold method was used for differentiating gas cells and non-cells (Crowley et al., 2000). Analysis was carried out on two sub-images (1,001 × 1,393 pixels, field of view) selected within the bread slice. The crumb cell features recovered were number, area, perimeter, and gas cell to total area ratio.

Color was measured in three different points of bread crust and crumb through a Minolta CR-10 Camera (Ahrné et al., 2007). L*, a*, b* color space analysis method was used, where L* represents lightness (white-black) and a* and b* represent the chromaticity coordinates (red-green and yellow-blue, respectively).

Proximal analysis of bread was carried out as described above. The total soluble and insoluble dietary fiber content of bread was determined by the enzymatic-gravimetric AOAC Official Method 991.43. Total fructans content was measured in triplicate using the Megazyme Fructan HK Assay kit (Megazyme International Ireland Ltd., Wicklow, Ireland) according to the principles described by the AOAC Method 999.03 and the AACC Method 32.32. Extraction and determination of fructans were carried out according to the manufacturer’s instructions.

### Sensory Analysis of Bread

Sensory analysis of bread was carried out by 10 panelists (5 male and 5 female panelists, aged between 25 and 35 years), according to the method described by Haglund et al. (1998) and Rizzello et al. (2010b). Crust darkness, crumb darkness, crumb elasticity, crumb softness, crust crispness, crumb dryness, sourness, bitterness, rancid, astringency, toasted, nutty, fruitiness, sweetness, fermented, and aroma intensity were considered as sensory attributes using a scale from 0 to 5.

### Statistical Analysis

For each condition, samples obtained from three independent experiments were analyzed in triplicate. Analysis was performed using the ANOVA test for multiple comparisons (one-way ANOVA followed by Tukey’s procedure at *P* < 0.05), using the statistical software, Statistica 8.0 (Statsoft). Descriptive sensory attributes were analyzed by principal component analysis (PCA), using a covariance matrix with the software Statistica 8.0, Windows.

## Results

### Proximate Analysis of Flours

Similar (*P* > 0.05) total carbohydrate levels were found in both flours (72.1 and 73.2 g for 100 g of flour) (Table 1). DSF showed higher (by 400%) (*P* < 0.05) total dietary fibers content compared with DWF, mainly represented by an insoluble fraction (94%) (Table 1). Fats content in DSF was significantly (*P* < 0.05) higher (by 600%) than DWF. On the contrary, DSF showed significantly (*P* < 0.05) lower water (by 37%) and protein (by 37%) contents than DWF (Table 1).

**TABLE 1:** Proximate analysis of durum wheat flour (DWF) and date seeds flour (DSF).

	DWF	DSF
Water (g per 100 g of flour)	15.0 ± 0.5^a^	9.5 ± 0.4^b^
Proteins (g per 100 g of flour)	10.0 ± 0.3^a^	6.4 ± 0.2^b^
Fats (g per 100 g of flour)	1.4 ± 0.1^b^	9.8 ± 0.1^a^
Total carbohydrates (g per 100 g of flour)	72.1 ± 1.4	73.2 ± 1.8
Total dietary fibers (g per 100 g of flour)	14.4 ± 0.5^b^	72.0 ± 0.5^a^
Soluble dietary fibers (g per 100 g of flour)	3.5 ± 0.2^b^	4.2 ± 0.5^a^
Insoluble dietary fibers (g per 100 g of flour)	11.0 ± 0.6^b^	67.8 ± 0.9^a^
Energy value (Kcal)	341 ± 8^b^	406 ± 9^a^

*^a,b^Means within the rows with different superscript letters are significantly different (P < 0.05).*

### Isolation, Typing, and Identification of Lactic Acid Bacteria and Yeasts

Lactic acid bacteria and yeasts were found in DSF at cell densities of 2.55 ± 0.51 and 2.54 ± 0.28 Log CFU/g, respectively. DSF dough had an initial pH value of 5.68. During the propagation of sourdoughs, cell densities of lactic acid bacteria and yeasts increased (*P* < 0.05) in all the sourdoughs. Lactic acid bacteria and yeasts increased (*P* < 0.05) to 7.68 ± 0.12 and 7.82 ± 0.10 Log CFU/g, respectively, and the pH decreased (*P* < 0.05) to 4.80 ± 0.06 in mature (after 8 refreshments) type I DSF sourdough. A similar trend was observed for mature sourdough of DSF added with sugars, with lactic acid bacteria and yeasts at cell densities of 7.64 ± 0.11 and 7.99 ± 0.09 Log CFU/g, respectively, and a final pH of 4.69 ± 0.04. As expected, the addition of yeast extract or yeast extract plus sugar further promoted (*P* < 0.05) the growth of lactic acid bacteria (8.80 ± 0.09 and 8.62 ± 0.10 Log CFU/g, respectively) and yeasts (7.98 ± 0.23 and 8.24 ± 0.16 Log CFU/g, respectively). The lowest (*P* < 0.05) pH value (4.29 ± 0.03) was found in DSF sourdough added with yeast extract plus sugar, whereas the addition of only yeast extract had no decreasing effect on the final pH (4.92 ± 0.02).

Presumptive lactic acid bacteria were isolated and identified by partial sequencing of the 16S rRNA and were biotyped through RAPD-PCR analysis. Fifty-seven isolates of lactic acid bacteria were identified as *Leuc. mesenteroides* (42 isolates) or *L. plantarum* (15). Based on RAPD patterns, 36 isolates were grouped into 7 clusters at the similarity level of 82.5%, with 21 unclustered isolates (Supplementary Figure 1). *L. plantarum* strains were isolated only from DSF sourdough added with yeast extract plus sugar, whereas *Leuc. mesenteroides* strains were found in DSF and in all mature sourdoughs.

Yeasts were also isolated and identified by partial sequencing of the 26S rRNA and were biotyped through RAPD-PCR analysis. Fifty-three isolates of yeasts were identified as *Pichia kudriavzevii* (17 isolates), *Saccharomyces cerevisiae* (28), *Wickerhamomyces subpelliculosus* (7), or *Rhodotorula mucilaginosa* (1). *R. mucilaginosa* and *W. subpelliculosus* strains were isolated only from DSF, *S. cereviasiae* strains were isolated only from doughs, whereas *P. kudriavzevii* strains were isolated from the dough and DSF. Based on RAPD patterns, 34 isolates were grouped into 2 clusters at the similarity level of 90%, with 19 unclustered isolates (Supplementary Figure 2).

### Starters Selection

**FIGURE 1:**
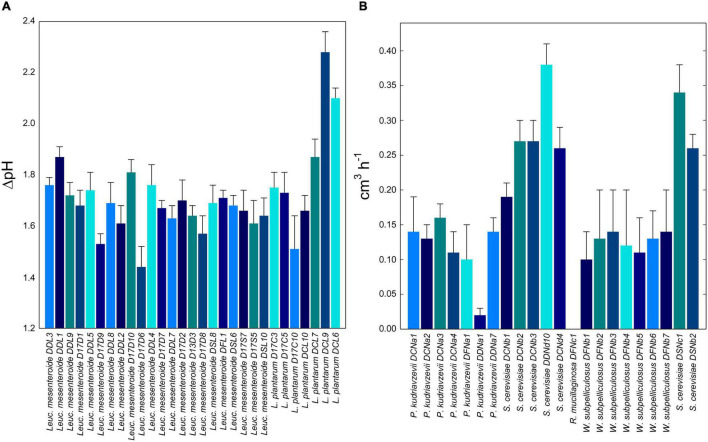
**(A)** Acidification capacities (difference in pH units between inoculation and the stationary phase, ΔpH) of 29 representative lactic acid bacteria strains during fermentation of date seeds flour dough. **(B)** Leavening performances (cm^3^/h) of 21 representative yeast strains during fermentation of date seeds flour dough. Date seeds flour (20%, wt/wt of flour) and durum wheat flour (80%, wt/wt of flour) were mixed with tap water to prepare date seeds flour doughs (dough yield of 174). Lactic acid bacteria or yeast strains were inoculated to a final cell density corresponding to *ca.* 7.0 Log CFU/g. Doughs were fermented at 30°C for 13.5 h.

Autochthons starter lactic acid bacteria and yeasts were selected based on their acidification kinetics and leaving power in DSF and dough, respectively. During fermentation, acidification was determined for all lactic acid bacteria. The ΔpH value ranged from 1.44 ± 0.08 to 2.28 ± 0.08 depending on the strains. *L. plantarum* DCL9 and *Leuc. mesenteroides* DDL1 were selected as the best facultative and obligatory heterofermentative strains (Figure 1A). Fifteen *Leuc. mesenteroides* strains showed a marked ability to synthesize EPS, including DDL1 strain, while no *L. plantarum* strains showed colonies with a mucoid appearance.

Regarding the leavening power, the highest values were found for *S. cerevisiae* strains, with DDNd10 and DSNc1 as the best (*P* < 0.05) performing ones (0.38 ± 0.03 and 0.34 ± 0.04 cm^3^/h, respectively). The yeast strains with the lowest leavening power were *R. mucilaginosa* DFNc1 and *P. kudriavzevii* DDNa1 (Figure 1B). Based on these results, *Leuc. mesenteroides* DDL1, *L. plantarum* DCL9, and *S. cerevisiae* DSNc1 and DDNd10 were selected and combined into a mixed starter for type I sourdough fermentation. Two strains of *S. cerevisiae* were combined according to the common practice of using multiple selected strains to reproduce the natural sourdough fermentation (Arora et al., 2021).

### Microbiological, Chemical, and Functional Characterization of Mature Sourdough

Mature DWF/DSF sourdough showed the higher lactic acid bacteria cell density (9.27 ± 0.19 Log CFU/g), compared with DWF/DSF-baker’s yeast-fermented dough and DWF-baker’s yeast-fermented dough (5.19 ± 0.24 and 5.19 ± 0.24 Log CFU/g respectively). On the contrary, yeast cell density was higher in baker’s yeast control doughs (*ca.* 7.8 Log CFU/g) than that in the DWF/DSF sourdough (7.05 ± 0.24 Log CFU/g). Because of lactic acid bacteria metabolism, sourdough acidification inhibited *Enterobacteriaceae* that were counted at very low cell density values (<10 CFU/g), while it reached *ca.* 2.6 Log CFU/g in the baker’s yeast control d oughs.

The chemical characterization of the doughs was performed before and after the fermentation. DWF/DSF-sourdough showed the lowest pH and the highest TTA values (Table 2). Acidification is mainly a consequence of lactic and acetic acid synthesis, produced by lactic acid strains during sourdough fermentation (Table 2). Lactic and acetic acid concentrations and their molar ratio define the parameter fermentation quotient (FQ) whose optimal value is in the range of 3–9 (Spicher, 1983). FQ values were 4.4 ± 1.1 in DWF/DSF sourdough, 2.4 ± 1.1 in DWF/DSF-baker’s yeast-fermented dough, and 1.3 ± 0.7 in DWF-baker’s yeast-fermented dough. The protein concentration of DWF/DSF unfermented dough was significantly (*P* < 0.05) lower than that in the DWF unfermented dough and DWF-baker’s yeast-fermented dough (Table 2). After fermentation, the concentration of proteins slightly decreased in DWF/DSF sourdough (by 13%) and DWF/DSF-baker’s yeast-fermented dough (by 12%), whereas no significant (*P* > 0.05) change was found for DWF-baker’s yeast-fermented dough. After fermentation, the concentration of total peptides significantly (*P* < 0.05) increased in DWF/DSF sourdough (by 17%), while it decreased in DWF/DSF-baker’s yeast-fermented dough and DWF-baker’s yeast-fermented dough (by 26 and 15%, respectively) (Table 2). No significant (*P* > 0.05) difference was found for TFAA content (Table 2).

**TABLE 2:** Chemical characterization of durum wheat flour unfermented dough (DWF unfermented dough), durum wheat flour-baker’s yeast-fermented dough (DWF-baker’s yeast-fermented dough), durum wheat flour/date seeds flour unfermented dough (DWF/DSF unfermented dough), durum wheat flour/date seeds flour type I sourdough (DWF/DSF sourdough), and durum wheat flour/date seeds flour baker’s yeast-fermented dough (DWF/DSF-baker’s yeast-fermented dough).

	pH	TTA (ml NaOH 0.1 M per 10 g)	Lactic acid (g per 100 g)	Acetic acid (g per 100 g)	Total proteins (g per 100 g)	Total peptides (g per 100 g)	Total free amino acids (g per 100 g)
DWF-unfermented dough	6.2 ± 0.2^a^	4.1 ± 0.1^d^	n.d.	n.d.	6.6 ± 0.4^a^	4.1 ± 0.1^b^	0.05 ± 0.02
DWF-baker’s yeast fermented dough	6.1 ± 0.2^a^	5.5 ± 0.2^c^	0.18 ± 0.06^b^	0.11 ± 0.07	6.4 ± 0.3^a^	3.5 ± 0.2^c^	0.05 ± 0.01
DWF/DSF-unfermented dough	6.0 ± 0.1^a^	6.2 ± 0.1^b^	n.d.	n.d.	5.2 ± 0.2^b^	4.2 ± 0.3^b^	0.03 ± 0.03
DWF/DSF-sourdough	4.0 ± 0.2^b^	13.3 ± 0.1^a^	1.46 ± 0.13^a^	0.23 ± 0.08	4.5 ± 0.4^c^	4.9 ± 0.2^a^	0.04 ± 0.01
DWF/DSF-baker’s yeast fermented dough	5.9 ± 0.2^a^	6.2 ± 0.1^b^	0.24 ± 0.09^b^	0.09 ± 0.07	4.6 ± 0.4^c^	3.1 ± 0.1^c^	0.05 ± 0.02

*^a,b,c^Means within the columns with different superscript letters are significantly different (P < 0.05).*

**FIGURE 2:**
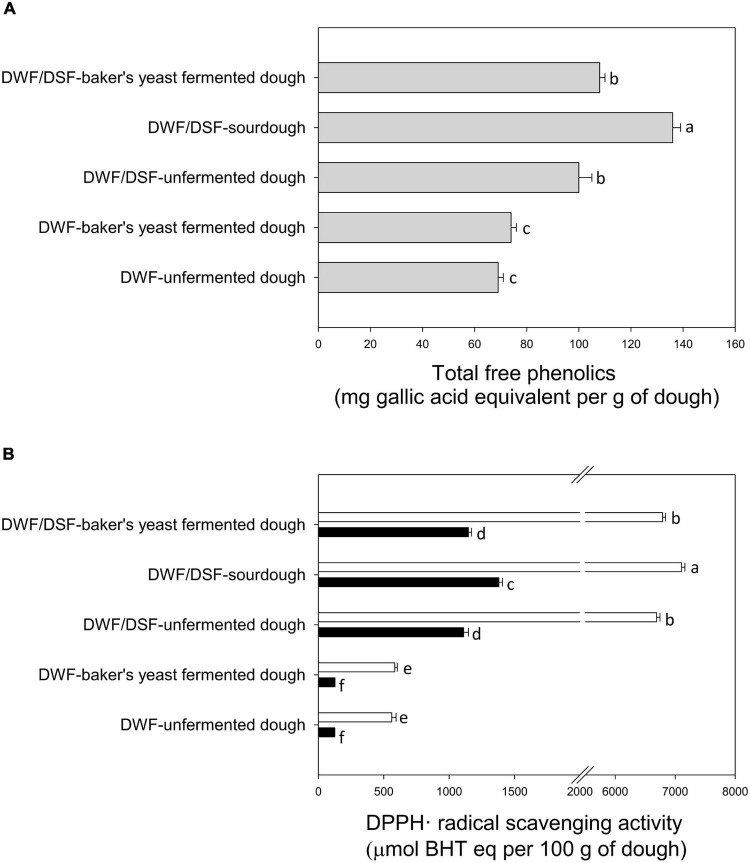
**(A)** Total free phenolics in durum wheat flour unfermented dough (DWF unfermented dough), durum wheat flour -baker’s yeast-fermented dough (DWF-baker’s yeast-fermented dough), durum wheat flour/date seeds flour unfermented dough (DWF/DSF-unfermented dough), durum wheat flour/date seeds flour sourdough (DWF/DSF-sourdough), and durum wheat flour/date seeds flour–baker’s yeast-fermented dough (DWF/DSF-baker’s yeast-fermented dough). **(B)** DPPH^⋅^ radical scavenging activity of water- (black bars) and methanol- (white bars) soluble extracts obtained from durum wheat flour unfermented dough (DWF unfermented dough), durum wheat flour–baker’s yeast-fermented dough (DWF-baker’s yeast-fermented dough), durum wheat flour/date seeds flour unfermented dough (DWF/DSF unfermented dough), durum wheat flour/date seeds flour sourdough (DWF/DSF sourdough), and durum wheat flour/date seeds flour–baker’s yeast-fermented dough (DWF/DSF-baker’s yeast-fermented dough). Data are the means ± standard deviation of three independent experiments analyzed in triplicate. Bars with different superscript letters differ significantly (*P* < 0.05).

DWF/DSF sourdough was characterized by the highest levels of total free phenolic compounds, followed by DWF/DSF unfermented dough and DWF/DSF-baker’s yeast-fermented dough (Figure 2A).

Antioxidant properties were assessed by determining the scavenging activity of MSE and WSE toward the DPPH^⋅^ radical. Apart from the type of sample, the type of solvent markedly affected the extraction of antioxidant molecules. As shown in Figure 2B, higher (*P* < 0.05) values of radical scavenging activity were obtained with MSE compared with WSE. The highest activity was detected in MSE from DWF/DSF sourdough (Figure 2B).

### Technological, Proximal, and Sensory Characterization of Bread

After baking, the nutritional, technological, and sensory features of the bread were investigated. The use of DSF (20%, w/w of flour) for DWF substitution in sourdough bread caused a significant (*P* < 0.05) increase in water, fats, and dietary fiber content and a decrease in the levels of total carbohydrates and proteins (Table 3). Sourdough biotechnology increased significantly (*P* < 0.05) the solubility of dietary fiber (Table 3). No significant (*P* > 0.05) difference was found for the energy value of bread (Table 3).

**TABLE 3:** Nutritional characterization of durum wheat flour-baker’s yeast bread (DWF-BYB), durum wheat flour/date seeds flour type I sourdough bread (DWF/DWF-SB), and durum wheat flour/date seeds flour-baker’s yeast bread (DWF/DSF-BYB).

	Water (g per 100 g)	Fat (g per 100 g)	Proteins (g per 100 g)	Total carbohydrates (g per 100 g)	Total dietary fiber (g per 100 g)	Soluble dietary fiber (g per 100 g)	Insoluble dietary fiber (g per 100 g)	Energy value (kcal per 100 g)
DWF-BYB	34.7 ± 0.8^b^	0.5 ± 0.1^b^	8.6 ± 0.4^a^	54.8 ± 1.0^b^	8.7 ± 0.2^b^	2.3 ± 0.1^a^	6.4 ± 0.1^c^	258 ± 3
DWF/DSF-SB	37.0 ± 1.0^a^	1.6 ± 0.1^a^	7.5 ± 0.2^b^	52.5 ± 1.2^a^	16.2 ± 0.2^a^	2.4 ± 0.1^a^	13.6 ± 0.2^b^	254 ± 3
DWF/DSF-BYB	37.4 ± 1.1^a^	1.6 ± 0.1^a^	7.4 ± 0.1^b^	52.1 ± 0.9^a^	16.3 ± 0.3^a^	1.6 ± 0.1^b^	14.7 ± 0.1^a^	253 ± 4

*^a,b,c^Means within the columns with different superscript letters are significantly different (P < 0.05).*

In terms of technological properties, DSF integration slightly (*P* > 0.05) affected the specific volume of bread (Table 4). The crumb structure of bread was evaluated by image analysis technology. Digital images were preprocessed to estimate crumb cell total area through a binary conversion (Supplementary Figure 3). As shown in Table 4, the cell total area (corresponding to the black pixel total area) was significantly (*P* < 0.05) higher in DWF/DSF-SB compared with the other two bread types. Crust and crumb color analysis of DWF/DSF-SB and DWF/DSF-BYB revealed lower (*P* < 0.05) lightness (L*) values than DWF-BYB (Table 4).

**TABLE 4:** Physical characterization of durum wheat flour-baker’s yeast bread (DWF-BYB), durum wheat flour/date seeds flour type I sourdough bread (DWF/DWF-SB), and durum wheat flour/date seeds flour-baker’s yeast bread (DWF/DSF-BYB).

	Specific volume (cm^3^ g^–1^)	Black pixel area (%)	Color indexes of crust			Color indexes of crumb		
			L*	a*	b*	L*	a*	b*
DWF-BYB	1.7 ± 0.2	34.9 ± 0.3^c^	63.5 ± 0.7^a^	6.7 ± 0.3^c^	26.4 ± 0.6^a^	63.4 ± 0.6^a^	0.9 ± 0.4^c^	19.8 ± 2.9^a^
DWF/DSF-SB	1.4 ± 0.1	44.9 ± 0.3^a^	48.2 ± 1.9^b^	8.3 ± 0.3^b^	15.9 ± 0.6^bc^	36.5 ± 3.8^b^	5.9 ± 0.4^b^	12.2 ± 1.3^b^
DWF/DSF-BYB	1.5 ± 0.3	42.3 ± 0.1^b^	49.5 ± 1.7^b^	10.2 ± 0.5^a^	18.0 ± 0.7^b^	38.7 ± 9.4^b^	7.2 ± 0.2^a^	11.6 ± 1.4^b^

*^a,b,c^Means within the columns with different superscript letters are significantly different (P < 0.05).*

**FIGURE 3:**
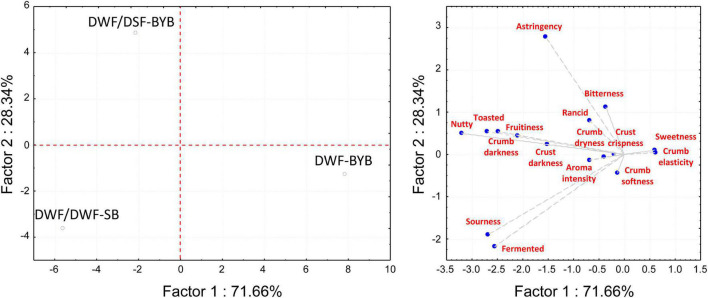
Principal component analysis (PCA) biplot, based on sensory analysis data of durum wheat flour baker’s yeast bread (DWF-BYB), durum wheat flour/date seeds flour sourdough bread (DWF/DWF-SB), and durum wheat flour/date seeds flour baker’s yeast bread (DWF/DSF-BYB). Sensory attributes were: crust darkness, crumb darkness, crumb elasticity, crumb softness, crust crispness, crumb dryness, sourness, bitterness, rancid, astringency, toasted, nutty, fruitiness, sweetness, fermented, and aroma intensity.

According to the sensory evaluation of bread, DWF/DSF-SB and DWF/DSF-BYB had in common some attributes (crust and crumb darkness, toasted, nutty, fruitiness; Figure 3). On the contrary, the sourdough biotechnology strongly affected the attributes related to taste (Figure 3). Some negative perceptions associated with DSF, like astringency, bitterness, and rancidity, were predominantly in DWF/DSF-BYB bread (Figure 3), where the highest scores for sourness and fermented notes were attributed to DWF/DSF-SB (Figure 3).

## Discussion

Sourdough fermentation was widely described as an effective tool to improve the technological and sensory features of unconventional flours (Gobbetti et al., 2020; Arora et al., 2021). Nevertheless, commercial starters or the traditional type I sourdough propagated in bakeries are tailor-made for the fermentation of wheat or rye flours. *De novo* assembling of sourdoughs by using non-conventional flours may benefit from starter-assisted fermentation to accelerate the establishment of an adapted and performing microbiota (De Vuyst et al., 2014). Consequently, we isolated autochthonous lactic acid bacteria and yeasts from DSF and DSF-derived doughs to build up a reservoir of strains from which to select starters ensuring rapid adaptation and high ecological fitness. *L. plantarum* and *Leuc. mesenteroides* were the only two lactic acid bacteria species identified. The experimental design we adopted for the isolation allowed us to hypothesize that *Leuc. mesenteroides* is well adapted to growth both on DSF and under the selective conditions of mature DSF sourdough, which is characterized by low pH and sugar starvation. *Leuc. mesenteroides* is frequently identified in raw and fermented plant matrices and is part of the wheat sourdough consortium, despite the predominance of lactobacilli (Jung et al., 2011; De Vuyst et al., 2014; Nionelli et al., 2014a; Di Cagno et al., 2016; Coda et al., 2017). On the contrary, *L. plantarum* was detectable only in DSF sourdough added with yeast extract plus sugar, despite its widely reported metabolic versatility (Filannino et al., 2016). Although DSF is deficient in certain nutrients (e.g., fermentable sugars and proteins) compared with DWF, *L. plantarum* is expected to be well adapted to plant matrices, even hostile ones, through the capability to implement alternative substrates transport and metabolism, specific responses functionally, and the expression of general stress response genes (Filannino et al., 2016). We can speculate that the fortification of dough with sugar and yeast extract and the consequent strong acidification may justify the abundance of the acid-tolerant *L. plantarum* species only in the fortified dough. Among the other sourdough players, *R. mucilaginosa* and *W. subpelliculosus* yeasts dominated the DSF, to be replaced by *S. cereviasiae* during DSF dough fermentation. Instead, *P. kudriavzevii* persisted during all propagation steps. Worldwide, *S. cerevisiae* is the species most frequently retrieved from wheat sourdough and other fermented foods, and it is also added to sourdoughs as baker’s yeast to emphasize the leavening power (Gobbetti, 1998; Vrancken et al., 2010; Reale et al., 2013; De Vuyst et al., 2017; Palla et al., 2019; Arora et al., 2021). Besides, *P. kudriavzevii* regularly occurs in bakery sourdoughs (Vrancken et al., 2010; De Vuyst et al., 2017; Arora et al., 2021).

To assemble *de novo* prepared sourdough with *ad hoc* selected lactic acid bacteria and yeasts, the screening criteria may be varied, including technological, biochemical, and nutritional attributes (Palla et al., 2020; Reale et al., 2020). Nevertheless, acidification and leavening performances are the most screened, trying to speed up sourdough fermentation at both artisanal and industrial levels (Arora et al., 2021). The acidifying properties of sourdough are associated with lactic acid bacteria, whereas the leavening power is attributable to yeasts and, to a lesser extent, to lactic acid bacteria. Among the lactic acid bacteria species isolated, *L. plantarum* DCL9 and *Leuc. mesenteroides* DDL1 were selected based on the acidification rate. Both species were previously proposed as starters for sourdough fermentation (Montemurro et al., 2020; Arora et al., 2021). Although the performances of *L. plantarum* strains were higher compared with *Leuc. mesenteroides*, the choice was made to ensure both facultative and obligatory heterofermentative species. The rate of acidification reflects the adaptability of the strains to the matrix, and, at the same time, is important to limit colonization by pathogenic and spoilage microorganisms, other than affecting the sensory, textural, and nutritional properties of the bread (Montemurro et al., 2020; Arora et al., 2021). Specifically, the synthesis of acetic acid, mainly by obligate heterofermentative species, affects the fermentation quotient, which in turn has a positive effect on bread flavor (Montemurro et al., 2020; Arora et al., 2021). DSF contains high amounts of phenolic compounds (Hilary et al., 2020), which may impair the ecological fitness of relevant bacteria during fermentation (Sánchez-Maldonado et al., 2011; Sekwati-Monang et al., 2012; Filannino et al., 2014; Dinardo et al., 2019). *L. plantarum* species was previously reported as more tolerant against phenolics, especially hydroxycinnamic acids, compared with other sourdough-associated bacteria, including *Fructilactobacillus sanfranciscensis*, which is a well-known key lactic acid bacterium in wheat sourdough (Sekwati-Monang et al., 2012; Dinardo et al., 2019). The ability of *L. plantarum* to tolerate hydroxycinnamic acids was previously explained by its ability to metabolize such compounds through decarboxylation and/or reduction reactions, which have been hypothesized to underlie both a detoxification mechanism and the maintenance of energy balance (Sánchez-Maldonado et al., 2011; Filannino et al., 2015; López de Felipe et al., 2021). *Leuc. mesenteroides* also revealed a fair tolerance to hydroxycinnamic acids, although no information is available on the implementation of specific adaptation mechanisms by strains of this species (Filannino et al., 2014). Out of 22 yeast strains, those with the greatest leavening power all belonged to *S. cerevisiae* species, thus confirming the high suitability of such yeast for the preparation of baked goods, also containing non-wheat flours like DSF (Palla et al., 2019).

The use of mixtures of strains, each one selected for a different biochemical and/or technological trait, was previously proved effective to get optimal sourdough fermentation (Rizzello et al., 2016; Nionelli et al., 2018; Montemurro et al., 2019). Thus, we assembled *Leuc. mesenteroides* DDL1, *L. plantarum* DCL9, and *S. cerevisiae* DSNc1 and DDNd10 in a mixed starter to be inoculated for obtaining a mature type I sourdough after consecutive refreshments, in which a fraction of the DWF was replaced by DSF. Outcomes of fermentation processes depend on specific parameters (e.g., dough yield, percentage of DSF, and length of fermentation), which we optimized and collected in an *ad hoc* protocol to obtain a standardised and agreeable sourdough bread fortified with DSF. As expected, the integration of DWF with the DSF led to increased content of total phenolic compounds. In addition, sourdough fermentation caused a substantial increase of free phenolics amount, which may result in more bioavailable forms to humans than the bound form (Roasa et al., 2021). The increased level of the free fraction likely accounts for the strong radical scavenging activity of MSE obtained from DWF/DSF sourdough and can be traced back to specific enzymatic activities held by lactic acid bacteria and yeasts, which can act individually or in a complementary way (Canonico et al., 2016; Boudaoud et al., 2021; Tlais et al., 2021). For instance, microbial esterases and glycosyl hydrolases can release phenolics from their conjugated form (Gänzle, 2020; Acin-Albiac et al., 2021; Tlais et al., 2021). Furthermore, phenolic acids may be metabolized and converted into reduced or decarboxylated derivatives (Sánchez-Maldonado et al., 2011; Santamaría et al., 2018a,b; Gaur et al., 2020), which may exert higher antioxidative, immunomodulatory, and antimutagenic properties compared with their precursors (Nićiforović and Abramovič, 2014; Senger et al., 2016; Septembre-Malaterre et al., 2018). The ability to metabolize phenolic acids has been found both in lactobacilli like *L. plantarum*, and yeasts like *S. cerevisiae* (Rodríguez et al., 2008; Santamaría et al., 2018a,b; Gaur et al., 2020; Boudaoud et al., 2021).

The integration of DWF with the DSF led to an increased level of dietary fiber of the dough. The total contents did not show significant variations before and after fermentation, but the ratio between insoluble and soluble fibers decreased during sourdough fermentation due to the acidification and enzymatic hydrolysis, as previously shown for other cereal- and pulses-based matrices (Nionelli et al., 2014b; Verni et al., 2020).

The production of fortified bakery products avoiding any deterioration of sensory features is one of the main challenges for researchers and the food industry. The fermentation quotient of DWF/DSF sourdough was within the recommended range to positively affect the flavor and textural properties of doughs (Spicher, 1983). Usually, the high concentration of fibers is associated with the weak dough structure and decreased bread volume (Wang et al., 2002). According to the specific volume and crumb structure of DWF/DSF-SB, our *ad hoc* setup protocol seemed to minimize the structural negative effects attributable to the DSF integration. DSF is also rich in tannins which impart bitterness and astringency perception to the fortified bread and reduce protein digestibility and iron uptake (Echegaray et al., 2021). Based on sensory evaluation scores, sourdough fermentation strongly reduced the perceptible astringency and bitterness. This is likely attributable to microbial tannases, which release gallic acid from tannins, thus reducing their interaction with proteins and their affinity for iron (Gänzle, 2020). Extracellular and intracellular tannases were previously described in *L. plantarum* and *S. cerevisiae* (Iwamoto et al., 2008; Curiel et al., 2009; Jiménez et al., 2014; Hawashi et al., 2019).

## Conclusion

This study aimed to propose DSF as an innovative ingredient for sourdough bread production through a sustainable bio-recycling. The screening among autochthonous strains allowed overcoming some limiting factors to microbial growth, such as the higher phenolics content of DSF compared with DWF. The mixed starter, consisting of selected *Leuc. mesenteroides*, *L. plantarum*, and *S. cerevisiae* strains, allowed to obtain a mature type I sourdough after consecutive refreshments, in which a fraction of the DWF was replaced by DSF. Sourdough biotechnology was confirmed as a suitable procedure to improve some functional and sensory properties of DWF/DSF mixture formulation. The radical scavenging activity increased due to the consistent release of free phenolics. Perceived bitterness and astringency were considerably diminished, likely because of tannin degradation. In conclusion, our study provides a realistic option that combines the exploitation of agro-food by-products and the manufacturing of palatable functional foods.

## Data Availability Statement

The datasets presented in this study can be found in online repositories. The name of the repository and accession numbers can be found below: GenBank (https://www.ncbi.nlm.nih.gov/genbank/)—OM731650-OM731662 and OM731665-OM731670.

## Author Contributions

HA, VC, and IC carried out the experiments. HA, VC, and ON performed the data analyses and prepared the figures and the tables. PF, IC, and RD conceived the study. PF administrated the project. HA and VC wrote the draft of the manuscript. PF, MG, and RD critically revised the manuscript. RD supervised the project. All authors read and approved the final manuscript.

## Conflict of Interest

The authors declare that the research was conducted in the absence of any commercial or financial relationships that could be construed as a potential conflict of interest.

## Publisher’s Note

All claims expressed in this article are solely those of the authors and do not necessarily represent those of their affiliated organizations, or those of the publisher, the editors and the reviewers. Any product that may be evaluated in this article, or claim that may be made by its manufacturer, is not guaranteed or endorsed by the publisher.
