# Supplementary material for: Date Seeds Flour Used as Value-Added Ingredient for Wheat Sourdough Bread: An Example of Sustainable Bio-Recycling
Source: Front Microbiol. 2022 Apr 18;13:873432. doi: 10.3389/fmicb.2022.873432 (PMC9062590; doi:10.3389/fmicb.2022.873432)
Supplement: Supplementary file 1 [file Presentation_1.pdf]

Supplementary Material

1. Supplementary Figures

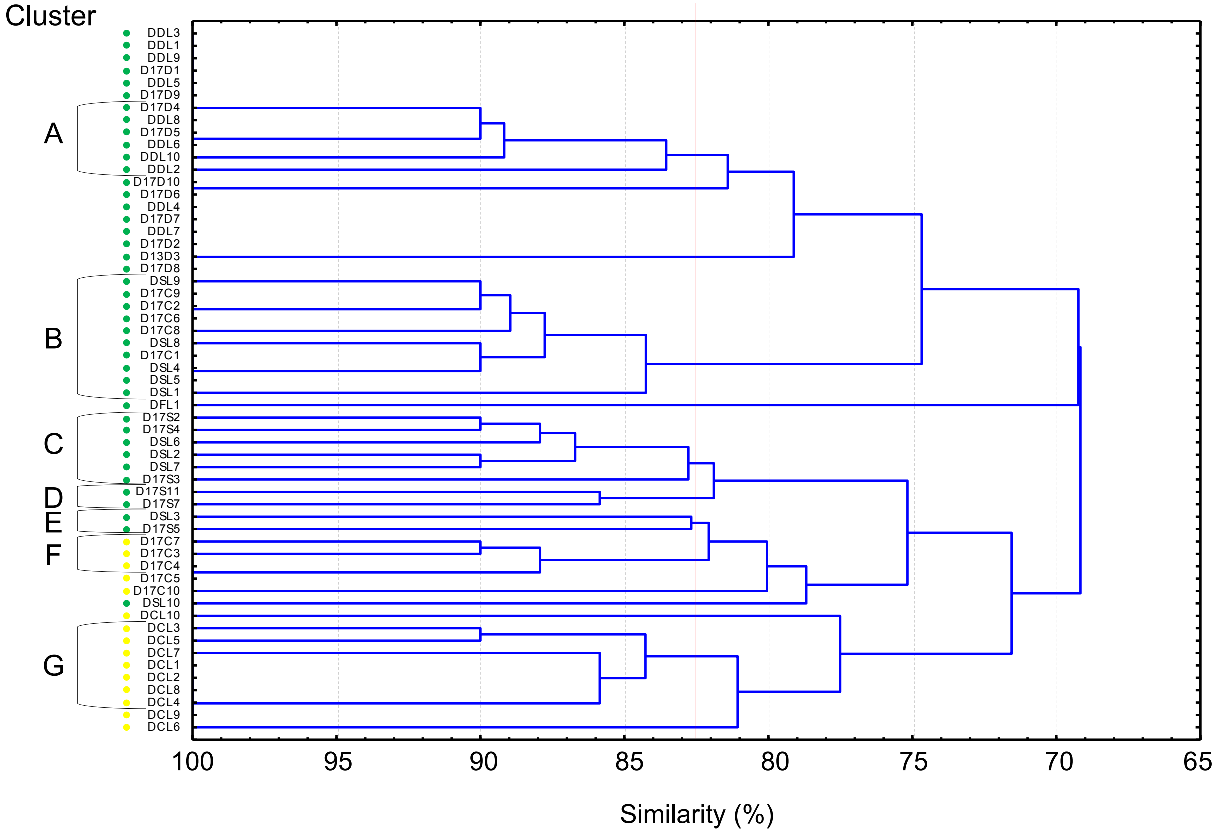

Supplementary Figure 1. Dendrogram of combined (primers P4, P7, and M13) RAPD profiles of lactic acid bacterium strains isolated from date seeds flour, date seeds flour dough fermented at 30 °C for 16 h, and date seeds flour type I sourdough. Cluster analysis was based on UPGMA algorithm. Clusters are indicated by capital letters A-G. Strains were identified as *Leuconostoc mesenteroides* (marked with green points) and *Lactiplantibacillus plantarum* (marked with yellow points).

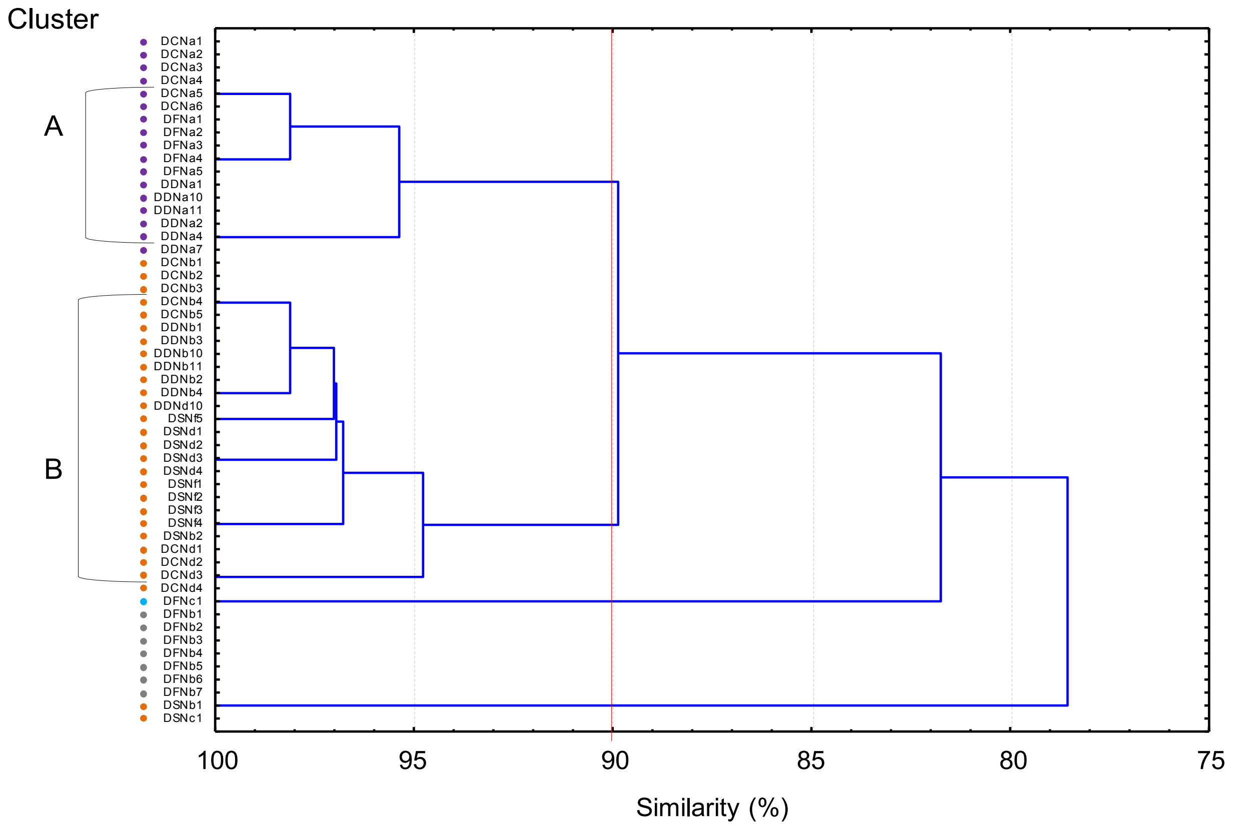

Supplementary Figure 2. Dendrogram of combined (primers M13m and RP11) RAPD profiles of yeast strains isolated from date seeds flour, date seeds flour dough fermented at 30 °C for 16 h, and date seeds flour type I sourdough. Cluster analysis was based on UPGMA algorithm. Clusters are indicated by capital letters A-B. Strains were identified as *Pichia kudriavzevii* (marked with violet points), *Saccharomyces cerevisiae* (marked with orange points), *Rhodotorula mucilaginosa* (marked with a cyan point), and *Wickerhamomyces subpelliculosus* (marked with gray points).

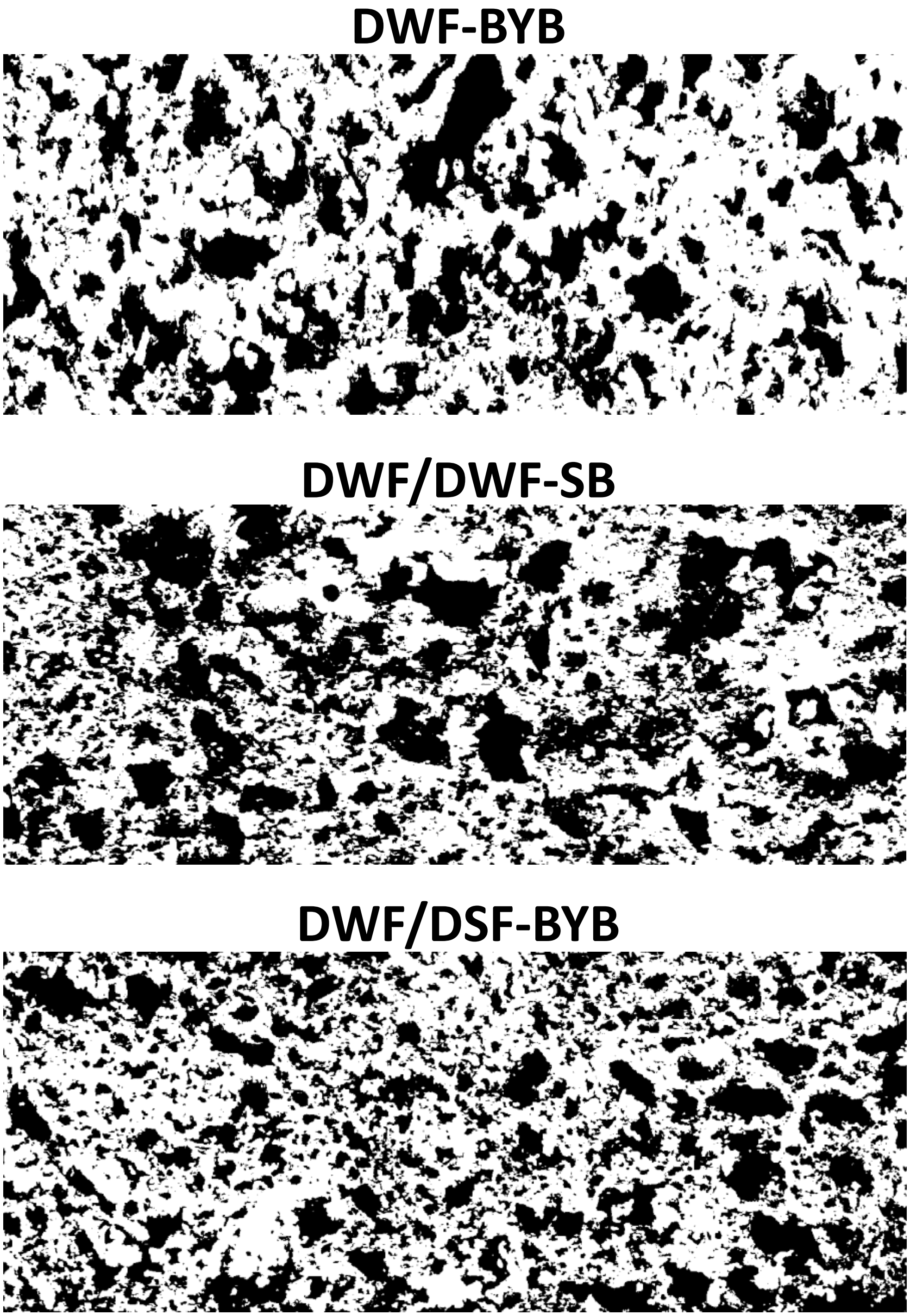

Supplementary Figure 3. Representative digital images of sections of durum wheat flour baker's yeast bread (DWF-BYB), durum wheat flour/date seeds flour sourdough bread (DWF/DWF-SB), and durum wheat flour/date seeds flour baker's yeast bread (DWF/DSF-BYB).
